# Integrated care for resected early stage lung cancer: innovations and exploring patient needs

**DOI:** 10.1136/bmjresp-2016-000175

**Published:** 2017-06-12

**Authors:** Jan Ho, Annette McWilliams, Jon Emery, Christobel Saunders, Christopher Reid, Suzanne Robinson, Fraser Brims

**Affiliations:** 1 Department of Respiratory Medicine, Sir Charles Gairdner Hospital, Nedlands, Western Australia, Australia; 2 Department of Respiratory Medicine, Fiona Stanley Hospital, Murdoch, Western Australia, Australia; 3 School of Medicine and Pharmacology, University of Western Australia, Perth, Western Australia, Australia; 4 General Practice and Primary Health Care Academic Centre, The University of Melbourne, Carlton, Victoria, Australia; 5 Department of Surgery, Fiona Stanley Hospital, Perth, Western Australia, Australia; 6 School of Public Health, Faculty of Health Sciences, Curtin University, Perth, Western Australia, Australia; 7 Curtin Medical School, Faculty of Health Sciences, Curtin University, Perth, Western Australia, Australia

**Keywords:** carcinoma, non-small cell; thoracotomy; evaluation health services, primary health care

## Abstract

There is no consensus as to the duration and nature of follow-up following surgical resection with curative intent of lung cancer. The integration of cancer follow-up into primary care is likely to be a key future area for quality and cost-effective cancer care. Evidence from other solid cancer types demonstrates that such follow-up has no adverse outcomes, similar health-related quality of life, high patient satisfaction rates at a lower cost to the healthcare system. Core elements for successful models of shared cancer care are required: clear roles and responsibilities, timely effective communication, guidance on follow-up protocols and common treatments and rapid routes to (re)access specialist care. There is thus a need for improved communication between hospital specialists and primary care. Unmet needs for patients with early stage lung cancer are likely to include psychological symptoms and carer stress; the importance of smoking cessation may frequently be overlooked or underappreciated in the current hospital-based follow-up system. There is therefore a need for quality randomised controlled trials of patients with resected early stage lung cancer to establish optimal protocols for primary care-based follow-up and to more adequately address patients' and carers' unmet psychosocial needs, including the crucial role of smoking cessation.

## Introduction

Lung cancer is the most commonly diagnosed cancer worldwide,^1^ and surgery offers the best chance of cure or long-term survival. There are variable early stage lung cancer surgical rates across developed countries of between 5% and 21%.^2^ Patients with resected early stage lung cancer have recurrence rates of between 30% and 75%,^3^ and it is likely that with increasing interest in and adoption of screening for lung cancer,^4^ rates of those having surgery for early stage disease may increase. In addition, patients with a previous lung cancer are at high risk of developing metachronous lung cancers at a rate of 1%–5% per annum.^5^

There is no consensus as to the duration and nature of follow-up following curative surgical resection of lung cancer with the most recent American guidelines citing the evidence base as ‘weak, low quality’.^6^ Typically, after postoperative follow-up, current practice involves CT of the chest at 6 months and then annually up to 5 years after resection. The American guidelines recommend every 6 months for 2 years,^6^ although there is a wide variation in practice in across countries with similar healthcare systems. This follow-up is normally hospital based, with respiratory specialists or thoracic surgeons. After lobectomy, local recurrence is most common in the first 2 years with the risk of recurrence nearly twofold higher (HR 1.86; 95% CI 1.01 to 3.41) in persistent smokers.^7^

Cost-effective healthcare relies on the provision of appropriate care in the right time at the right place; countries with a strong primary care component have been demonstrated to be more cost-effective than those that are over-reliant on hospital-led services.^8^ As cancer survival rates increase, the focus on managing patient flow back into primary care is a key area for future-effective and cost-effective cancer care.^9^ It is recognised that follow-up of patients undergoing palliative surgical interventions is very different; these patients are likely to benefit from regular secondary care contact.^10^ This article will concentrate on follow-up after surgery with curative intent for non-small cell lung cancer (NSCLC).

### The role of primary care in follow-up of patients with cancer

There is increasing acceptance of the role of primary care in the follow-up of cancer survivors,^11–14^ and in turn this is seen as increasingly critical for the long-term sustainability for healthcare systems in many developed countries.^15^ In many developed countries, there is an increasing financial demand on secondary and tertiary care services. There is thus a need to better define which clinical services require specialist oversight and which conditions could, perhaps with appropriate guidance and oversight,^16 17^ be acceptably managed within primary care.

Evidence from randomised clinical studies in bowel and breast cancer follow-up suggests that integrated follow-up in primary care has no adverse outcomes, similar health-related quality of life and high patient satisfaction rates.^18 19^ The results of an Australian randomised control trial examining integrated care with hospitals and general practitioners (GPs) for patients with prostate cancer also demonstrated that this was a safe and acceptable model of care at lower cost to the healthcare system.^20^ GPs feel confident and enabled to contribute to long-term cancer care^21^ and cancer survivors are satisfied with care delivery in primary care.^22^

There have been a number of studies examining follow-up of lung cancer in different settings. A retrospective cohort study in Japan reported the outcomes of postoperative patients with NSCLC dependent on follow-up with either thoracic surgeons or chest physicians.^23^ Survival in more advanced stage disease was better with the chest physician group; there was data to suggest that the use of regular CT chest scans may improve detection of recurrence (or new primary) to enable therapy with curative intent (regardless of doctor in charge of follow-up).

Another retrospective cohort study from Canada describes outcomes of postsurgical patients with NSCLC, comparing thoracic surgeon followed by other health professionals.^24^ Despite hospital clinic follow-up, two-thirds of recurrences were detected by the patient’s GP, with no overall survival differences. It was postulated that GP-based care might be associated with 75% cost savings from follow-up. A randomised controlled trial from the UK comparing nurse-led care with conventional approaches for advanced NSCLC reported improved satisfaction, earlier recognition of deterioration, better emotional functioning, fewer consultations and no difference in survival with nurse-led care.^25^ Nurse-led care is reported to be acceptable to patients, carers, GPs and treating physicians.^26^ Such non-specialist follow-up may therefore be acceptable as long as there are clear protocols including the ability to refer to specialists easily and access to appropriate radiology.^26^

The potential shift in the care for patient with cancer with greater GP involvement also raises concerns that GP workloads may be increased.^21^ There is additional concern that perhaps GPs will see very few of these cases in their practice and thus may not be adequately confident or have sufficient experience to safely follow-up these patients.^15 27^ The proposed solution in this situation would be the use of a protocol in conjunction with clinical acumen to guide follow-up.^28^ Evidence suggests that certain core elements for successful models of shared cancer care are required: clear roles and responsibilities for GPs, timely effective communication about care, guidance on follow-up protocols and management of common treatment side effects and rapid routes to access specialist care.^16 17 20^ Therefore, a more collaborative and integrated approach would allow GPs to participate more with the follow-up care of patients with cancer, perhaps with integration with virtual or remote monitoring clinics from secondary care.

In some countries, access to specialist care varies, notably, specialist visits in populations that have a lower income or education levels are lower. The inequity in specialist access favours the populations with higher income and education in Organisation for Economic Co-operation and Development countries.^22 23 29 30^ Access to GPs, which is comparably better than access to specialists, would potentially improve this inequity and allow the same (or improved) standards of care.

### Unmet needs for patients with lung cancer

As acknowledged in the National Institute for Health and Care Excellence lung cancer clinical guidelines, there are sparse data examining unmet needs for patients with early stage lung cancer.^10^ Interviews with patients with lung cancer (with more advanced stage) have identified at least moderate levels of anxiety and depression.^31^ A further study reported that hospital consultants were failing to recognise anxiety in many patients and largely overlooked the needs of informal carers (in whom it was reported had the greatest onus of care).^32^ Key areas of unmet need may be most apparent during periods away from acute care.^32^ Other in depth interviews with patients with lung cancer and carers identified feelings of isolation and identified a need for coordinated family-oriented care.^33^ Patients identified a reliance on their hospital-based consultant and found it difficult to transition back to primary (or palliative) care. Variations in perceptions of care may be associated with a patients’ educational level, with a higher level of education associated with more focus on the logistics of care, rather than the psychosocial aspects.^34^

There is a further unmet need to support smoking cessation in lung cancer survivors with a 5-year survival rate following resection for lung cancer of 77% in those able to quit and 33% in those unable to quit smoking.^7^ Between our two institutions, 33%–62% of newly diagnosed lung cancer cases between 2015 and 2016 were current smokers at the time of their diagnosis (unpublished data). Physicians and surgeons who treat lung cancer give limited smoking cessation advice and implementation of plans due to limited resources.^35^ Smoking cessation and support for other behaviour change are key roles of primary care with links to community-based resources to support patients to stop smoking and adopt a healthier lifestyle.

Therefore, the present system of hospital-based care may not be adequately identifying all the needs of a patient with lung cancer, in particular, psychosocial aspects. There is further need for improved communication between hospital specialists and GPs^33 36 37^ and a more personalised explanation of treatment plan for patients.^38^ It is already accepted that primary care physicians are motivated and capable of providing follow-up to patients with cancer after their initial treatment, with good satisfaction among patients. Additionally, there is an identified economic need to decentralise care away from secondary and tertiary centres where possible.^15^

We contend that there is a need for quality randomised controlled trials of patients with resected early stage lung cancer to elucidate suitable patient populations for GP-led follow-up, development of protocols for optimal follow-up, more adequately address patients’ and carers’ unmet psychosocial needs and perform an economic evaluation of such an approach to guide future follow-up of these high-risk patients. There is further need for the development of a quality lung cancer registries (similar to the highly successful UK-based National Lung Cancer Audit database)^39^ that will not only lead to improved patient care but also may facilitate registry-based trials in the future.^40 41^

## Conclusion

Currently, there is inadequate data to inform the optimal care of patients who have had surgery for early stage lung cancer with curative intent. The current hospital-based system may not be addressing holistic patient-oriented care (including smoking cessation) adequately, together with the suboptimal use of secondary and tertiary hospital resources. High quality data and randomised studies are required to evaluate these key issues and improve care for these patients.
